# Separating sensitivity from exposure in assessing extinction risk from climate change

**DOI:** 10.1038/srep06898

**Published:** 2014-11-04

**Authors:** Maria G. Dickinson, C. David L. Orme, K. Blake Suttle, Georgina M. Mace

**Affiliations:** 1 Department of Life Sciences, Imperial College at Silwood Park, Ascot, Berkshire SL5 7PY, UK; 2 Grantham Institute, Imperial College London, Exhibition Road, South Kensington, London, SW7 2AZ; 3 Department of Genetics, Evolution and Environment, University College London, Gower Street, London WC1E 6BT

## Abstract

Predictive frameworks of climate change extinction risk generally focus on the magnitude of climate change a species is expected to experience and the potential for that species to track suitable climate. A species' risk of extinction from climate change will depend, in part, on the magnitude of climate change the species experiences, its exposure. However, exposure is only one component of risk. A species' risk of extinction will also depend on its intrinsic ability to tolerate changing climate, its sensitivity. We examine exposure and sensitivity individually for two example taxa, terrestrial amphibians and mammals. We examine how these factors are related among species and across regions and how explicit consideration of each component of risk may affect predictions of climate change impacts. We find that species' sensitivities to climate change are not congruent with their exposures. Many highly sensitive species face low exposure to climate change and many highly exposed species are relatively insensitive. Separating sensitivity from exposure reveals patterns in the causes and drivers of species' extinction risk that may not be evident solely from predictions of climate change. Our findings emphasise the importance of explicitly including sensitivity and exposure to climate change in assessments of species' extinction risk.

Climate change is a global anthropogenic threat and is expected to lead to shifts in the geographic distributions of species, ecological communities and even biomes. This global impact is already being seen, with shifts in species' distributions and life history timings evident from every continent and ocean and across most major taxonomic groups^1^. This has led many researchers to anticipate major species losses during the 21st century^2^,^3^. Targeting species for conservation before they are at imminent risk of extinction is necessary, but relies on early and accurate identification of species that will be at high risk^4^,^5^.

For other anthropogenic threats, such as habitat loss or overexploitation, extinction risk has been shown to depend only in part on the intensity of threat acting against the species; we refer to this as exposure to threat^6^. Exposure is extrinsic to the species and is primarily determined by geographic location. However, extinction risk also depends on the intrinsic aspects of a species' biology that determine its capability to withstand a given threat; we refer to this as sensitivity to threat. For a given threat, where species have the same threat exposure extinction risk is expected to be elevated in those species with higher sensitivity to that threat^7^. Consequently, variation between species in their sensitivity and exposure has been shown to an important predictor of patterns in extinction risk from a wide range of drivers^6^.

Predictions of extinction risk driven by climate change have typically focused on the level of species' predicted exposure to climate change. For example, extinction risk has been measured using the shift required in species' ranges to track suitable climates or the rate at which given climates are predicted to move across the globe or over time^3^,^8^,^9^. Whilst the magnitude of climate change a species experiences is undoubtedly an important component of its overall risk, the effects of a given level of exposure will be moderated by each species' intrinsic biological capability to withstand climate change, its sensitivity^5^,^7^,^10^.

Many drivers of threat, such as habitat loss or overexploitation, are focussed in particular habitats or species groups. Consequently, high exposure to threat may be a reasonable indicator of risk. In contrast, the global nature of climate change exposes a large number of species to a wide range of threat intensities. Understanding patterns in sensitivity to climate change and the extent to which high sensitivity and high exposure occur in the same species and regions is therefore likely to be even more critical to predicting patterns in climate change extinction risk than for other threats^5^,^10^.

Foden et al. (2013) present a broad assessment of exposure, sensitivity and adaptive capacity using qualitative scores. Here, we develop quantitative metrics at the species-level for both sensitivity and exposure to climate change. We base our measure of sensitivity simply on the range of conditions, along multiple climatic axes, encountered within the extent of occurrence of a species and representing a volume occupied within global climate space. We refer to this as the species' climate breadth: a species with a large climate breadth occupies an area covering a wide range of climates and consequently has low sensitivity. We develop a comparable measure for each species' exposure to future climate change based on the average predicted change (to 2050) in the same climate variables across each species' extent of occurrence (see Methods and Figure 1). We use these metrics to examine the extent to which high values of sensitivity and exposure to climate change co-occur, leading to elevated risk for individual species, and the extent to which explicit consideration of sensitivity separately from exposure may improve predictions of climate change impacts among species and across regions.

## Results

Species' climate breadth was only weakly positively correlated with exposure values. This finding was robust across all four relative concentration pathways (RCPs) (amphibians: *r* = 0.15–0.18, df = 4711; mammals *r* = 0.26–0.30, df = 4921; all *p* < 0.0001. Pearson's *r* calculated from species' mean sensitivity and exposure across greenhouse gas emission scenarios for each RCP). Species with high exposure are therefore not generally also those with small climate breadth, and hence show a wide range of sensitivities to climate change. Consequently, the number of species with both high sensitivity and high exposure to climate change is small (see Figure 2). Only around 1% of species (0.65–0.95% of mammals; 1.08–1.15% of amphibians across RCPs) fall in the 10% most extreme values for both sensitivity and exposure, rising to only around 3.5% (2.50–3.45% of mammals; 3.73–4.39% of amphibians) for the most extreme 20% of values and to around 20% (18.46–20.62% of mammals, 22.49–23.64% of amphibians) when the most sensitive and most exposed half of each taxon was considered.

Reflecting species level patterns, the geographical distribution of sensitive species differed from that of exposed species (Figure 3). There was significant positive correlation between grid cell averages of species' climate breadth and exposure values across RCPs (amphibians, *r* = 0.48–0.53, *p* < 0.03, ess = 15.1–20.2; Mammals, *r* = 0.54–0.56, *p* < 0.02, ess = 14.0–21.1). Regions with high exposure values were generally not those with small climate breadth, and hence high sensitivity to climate change. Amphibians showed insignificant or weak spatial correlation between the species richness of sensitive and exposed groups, with the largest *r* = 0.34 (*p* < 0.001, ess = 86.4) observed for RCP 2.6 with a 50% threshold. Mammals showed no significant correlation in richness patterns across all RCPs and for all thresholds between 10 and 50%.

In contrast, spatial patterns of sensitivity and of richness in sensitive species show strong positive correlations between amphibians and mammals: grid cell average climate breadth (*r* = 0.67, *ess* = 13.63, *p* = 0.0019) and sensitive species richness (10% threshold, *r* = 0.21, *ess* = 342.95, *p* < 0.0001; 20% threshold *r* = 0.30, *ess* = 342.95, *p* < 0.0001, 50% threshold *r* = 0.53, *ess* = 86.28, *p* < 0.0001).

## Discussion

Large magnitude changes in climate (high exposure) in a region do not necessarily imply an elevated extinction risk for the species that live there. Risk also depends on variation in species' intrinsic capabilities to tolerate changes in climate (their sensitivity). Regions or species where high sensitivity and high exposure to climate change co-occur would be expected to have a high level of extinction risk. Understanding patterns in the underlying drivers of extinction risk, sensitivity and exposure to climate change, allows more specific targeting of conservation interventions.

We find that global patterns in species' sensitivity to climate change are not congruent with patterns in exposure to climate change, measured equivalently across two major taxonomic groups. In line with previous small scale studies^12^,^13^, we find that species predicted to experience the greatest magnitudes of climate change (highest exposures) across their existing geographic distributions are in many cases those species expected to have relatively broad tolerances to climate so low sensitivity to climate change based on the range of climates they currently experience. We find that high sensitivity to climate change and high exposure to climate change, which together are expected to lead to high extinction risk, generally do not occur across the same species. Geographically, these species-level patterns are reflected as low overlap between regions of high sensitivity and regions of high exposure across the globe. Regions exposed to large magnitude changes in climate may not necessarily have high climate change-driven extinction risk if the species there have low sensitivity and are able to tolerate those changes. Similarly, regions exposed to small magnitude changes in climate may still be centres of extinction risk if species in those regions have high sensitivity and are unable to tolerate even small amounts of climate change.

We found that global patterns in sensitivity showed strong positive correlation between amphibians and mammals, suggesting cross-taxonomic hotspots of sensitivity to climate change. Such centres of sensitivity could be potential targets for conservation with benefits across multiple species groups, but may not be evident from patterns in predicted range change or predicted future climate.

Whilst low congruence between sensitivity and exposure is a potentially promising finding for biodiversity conservation, it emphasises the importance of understanding underlying patterns in sensitivity and exposure in predicting extinction risk from climate change. Examining sensitivity and exposure generates a more complex and informative global picture of climate change as a driver of extinction risk than assessment based on solely on predicted climate change. Our findings demonstrate quantitatively the additional information that can be gained from separating sensitivity to climate change from exposure to it. Methods for assessing extinction risk that concentrate on exposure almost entirely mask this information^3^,^14^,^15^,^16^,^17^.

We are not suggesting that measures of sensitivity and exposure should replace niche modelling based range shifts in assessments of climate change extinction risk, but that such measures are combined with existing conservation assessment and prioritisation systems to improve predictions and support conservation priority setting. Our capacity to make detailed, species-level predictions of climate change extinction risk is limited, in a large part, by lack of information on species' biology, ecology and life history^18^. Using what information we do have to extract as much understanding as possible of the patterns and drivers in climate change extinction risk is critical to maximising our ability to identify species most likely to be at risk. Combining measures of sensitivity and exposure with predictions of geographic range shift gives additional information on which to base conservation decision-making. For example, where predictions of future geographic range identify species as high risk, measures of sensitivity and exposure could highlight those species likely to be most vulnerable or most resilient and so help tailor conservation interventions or identify landscape targets such as the centres of sensitivity identified in this analysis^5^.

Our measures are not intended to be a full assessment of species' sensitivity and exposure to climate change. Biological traits, such as habitat or diet specialism, reliance on a specific biotic interaction or the degree of dependence on climatic cues to trigger life history events, will also affect a species' ability to withstand climate change^11^,^19^. Exposure will include not only climate change acting on the species, but also changes to species' habitats and communities as a second order effect of changing climate^2^. Since our projection of sensitivity rests solely on climatic niche, we assume that habitat structure remains relatively unaltered in the medium term. Recent work suggesting that plant responses to CO_2_ may lead to rapid change in ecosystem structure suggests that a broader definition of exposure may become necessary^20^.

Nor are our measures intended to be assessments of species' risk of extinction, as many other factors will contribute to risk. Variation in species' ability to adapt to change will also impact risk^11^,^21^. Genetic variability for any trait involved in sensitivity will affect species' ability to evolve and phenotypic plasticity for these traits will affect adaptation^21^. Some species will be more locally adapted to climatic conditions than others, with the potential for that local adaptation to increase their sensitivity to climate change^22^. Dispersal capabilities and reproductive capacity may also affect whether species can reach a new climatically suitable range and establish a viable population^11^,^19^. Ultimately, extinction risk will depend on the combination of sensitivity, exposure and adaptive capacity of species to climate change and other anthropogenic threats. Our measures are intended to form part of a flexible predictive framework of extinction risk which could also include biological traits affecting sensitivity or adaptive capacity and other measures of exposure to climate change (e.g. range shift, climate velocity or climate driven habitat change) along with other anthropogenic threats and any synergies between threats^23^,^24^.

Whilst our analysis shows the importance of separating sensitivity and exposure, there are some areas of uncertainty. Species that experience a wide range of climatic conditions were assumed to have an intrinsically lower sensitivity to changing climate than species that experience a narrow range of conditions. The distributions of many of the species included in this analysis are likely not solely limited by climate and the sensitivity of these species may be inflated by using geographic distribution to estimate climate breadth. However, whilst factors beyond climatic variables do constrain species distributions, when considered across a large number of species and multiple climatic variables, species' distributions should reflect general approximations of climatic tolerances for examining global level patterns^25^.

We assume the same climate variables to be important for all species to allow comparison between species. The importance of climate variables will vary between species and regions, so our climate breadths will be more representative for some species than others^26^. However, we used climate variables that are thought to be ecologically relevant and capture temperature and water limitation^14^,^15^,^17^,^27^,^28^. In any event, bioclimatic variables are derived from the same base temperature and precipitation data and are therefore likely have some relevance to all species, albeit at varying levels. A fuller assessment of realised niches, past, present and future, would obviously improve the climate breadth estimates, but these data do not exist for more than a handful of species at the moment^18^.

At a resolution of 1°, a large proportion of species, particularly amphibians, occur in a single grid cell. There is no variation in the climate data for a single cell, so these species must either be excluded from the analysis, as we have done here, or assigned a climate breadth of zero, which ignores diurnal and spatial variation in climate within the grid cell. Higher resolution climate data may allow measurement of climate breadths for some of these species. However, finer-scale analyses are likely to be misleading given the resolution of the underlying range maps^29^. Detailed distribution data, which may permit finer scale analysis, are simply not available for the majority of species. In any event, habitat suitability and biotic interactions, rather than climate, are thought to determine distributions at smaller spatial scales than those used here; finer resolution analysis may attribute to climate distribution patterns driven by these factors^30^.

As well as the methodological uncertainty described above, substantial uncertainty arises from the requirement to predict future climates. Whilst we present results based on the mean exposure value across 11 GCMs for each RCP, there is inevitable variation in species exposure values and resulting global patterns between GCMs. However, examining sensitivity separately from exposure decouples uncertainty as to future climate from uncertainty surrounding species' intrinsic abilities to cope with climatic change, which may be reduced by fuller understanding of species' sensitivities. As GCMs improve, model uncertainty in predicted climate may be reduced, but future climate will remain uncertain. Assessments can be revised as predictions of future conditions change or are better understood.

Despite these caveats, we use simple methods and data that are available for multiple species groups to show that high sensitivity and high exposure to climate change rarely occur in the same species. We demonstrate quantitatively that separating intrinsic biological patterns in sensitivity to climate change from extrinsic threat processes in predicting species' extinction risk from climate change provides additional information on which to base conservation decision making and maximises information extraction from the limited data available. We emphasise the need to move towards a flexible, integrated framework for assessing extinction risk from climate change.

## Methods

### Data

Species' range polygons (extents of occupancy) for terrestrial mammals and amphibians were obtained from the IUCN (http://www.iucnredlist.org/technical-documents/spatial-data, access dates: mammals 29/10/2009; amphibians 12/01/2010) and converted to presence-absence 1° latitude × 1° longitude grids in ArcGIS. A species was deemed to be present in a cell if any part of the cell was overlapped by the species' range.

Climate data were obtained from WorldClim (http://www.worldclim.org/download, access dates 20–30/05 2014). We used six bioclimatic variables: annual mean temperature, mean temperature of the warmest and coldest quarters to describe both heat and cold limitation and temperature seasonality; annual rainfall as total water availability; rainfall seasonality to describe variation in rainfall and rainfall in the warmest quarter to describe water limitation. These have been shown to be ecologically relevant and to limit amphibian and mammalian distributions^14^,^15^,^17^. To allow comparison between species and across taxonomic groups, we used the same climatic variables for all species. Climate data were at a resolution of 10 arc-minute, so were averaged within 1° grid cells to match the resolution of the species data.

**Figure 1:**
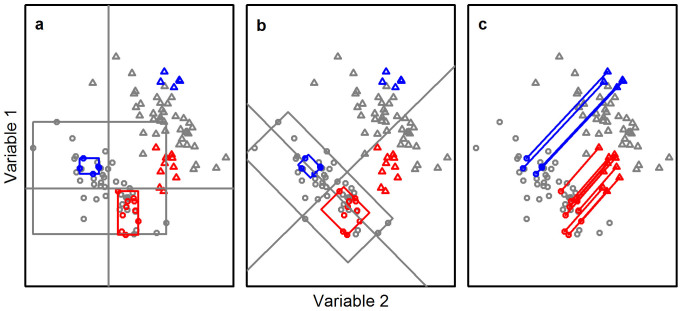
Schematic illustration of climate breadth and mean climate change. Two example climate variables are shown from six used. Climate variables within grid cells showing current values (circles) and predicted values after climate change (triangles). The cells occupied by two species are shown (blue, red points) for both current and predicted values. (a) The sensitivity of a species is defined by the volume of climate space corresponding to the species geographic range, which is derived from the width of the values experienced along each environmental axis (edge lengths of rectangles for each species' points). (b) Covariation may inflate the environmental width of those axes and so a rotation is used to produce orthogonal environmental axes for calculating sensitivity. (c) The exposure of a species is measured as the arithmetic mean of the Euclidean distances (blue, red lines) between the current and predicted environmental values of occupied cells. In this example, one species (blue) is sensitive and exposed, with a narrow environmental width and a high mean displacement across the cells in which it resides. In comparison, the second species (red) is neither sensitive nor exposed, having broad environmental widths and short environmental displacements.

**Figure 2:**
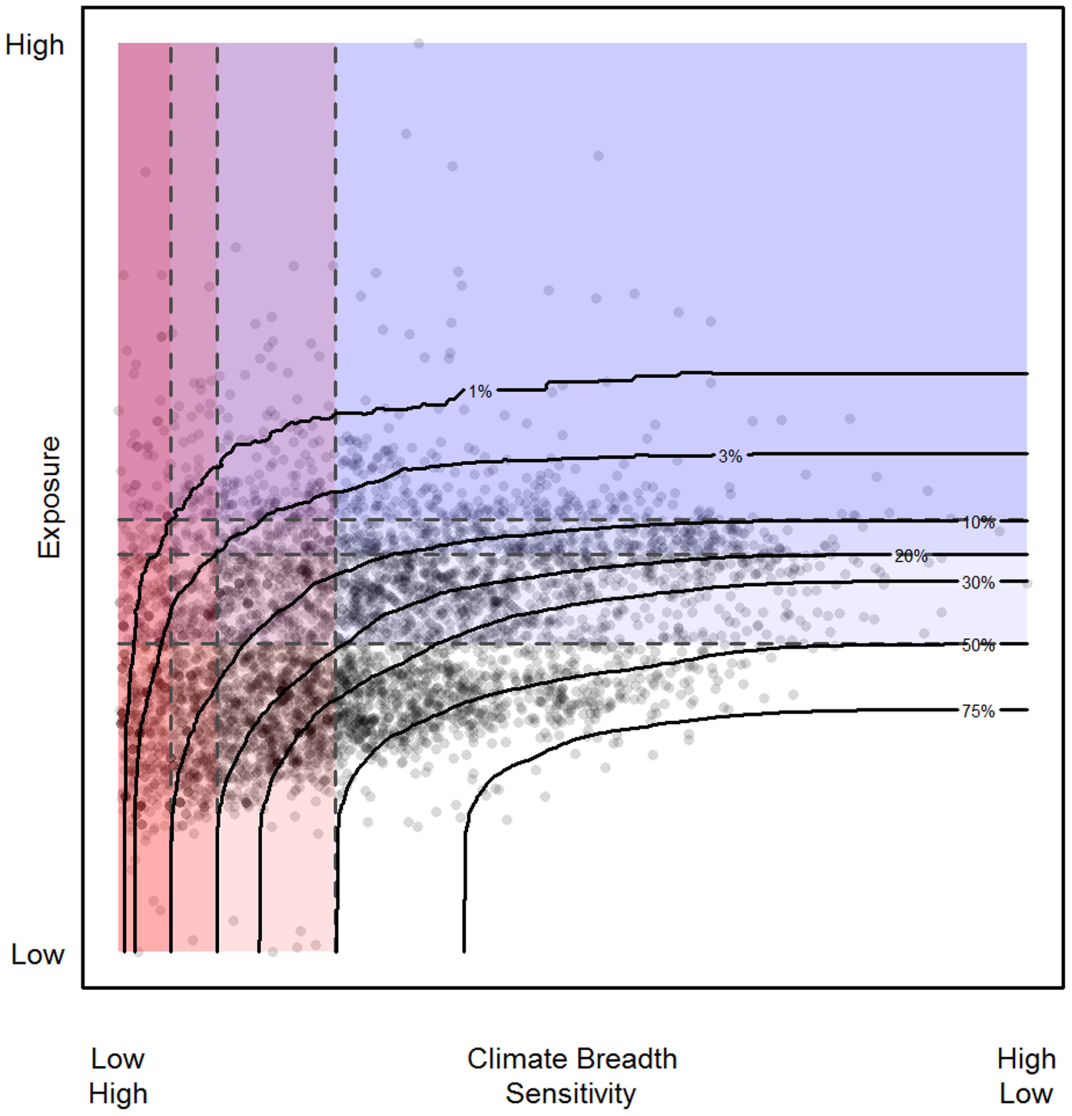
Identifying sensitive, exposed and at risk species. Mean climate breadth and exposure values across GCMs for both amphibians and mammals under RCP 4.5 Species (grey points) with high exposure may not be sensitive to climate change (blue region) and hence are better able to withstand climate change across their ranges. Species with narrow climate breadth may be sensitive to climate change but not exposed (red region) and their risk would need to be reassessed as estimates of climate change across their ranges are updated. We identify species as vulnerable when they are projected to experience a high magnitude of climate change which they are expected to be relatively unable to withstand (varying purple regions). Dashed horizontal and vertical lines define thresholds used to place species into broad categories of sensitivity and correspond to the most extreme 10, 20 and 50% of climate breadth (smallest values) and exposure values (highest values). The contour lines in black show the proportion of species that are both exposed and sensitive (falling in the region above and to the left) for different sensitivity and exposure thresholds. As examples, approximately 1% of species fall under the combination of the 10% exposure and sensitivity thresholds, approximately 3% of species fall under the combination of the 20% exposure and sensitivity thresholds and approximately 20% fall under the combination of 50% exposure and sensitivity thresholds.

**Figure 3:**
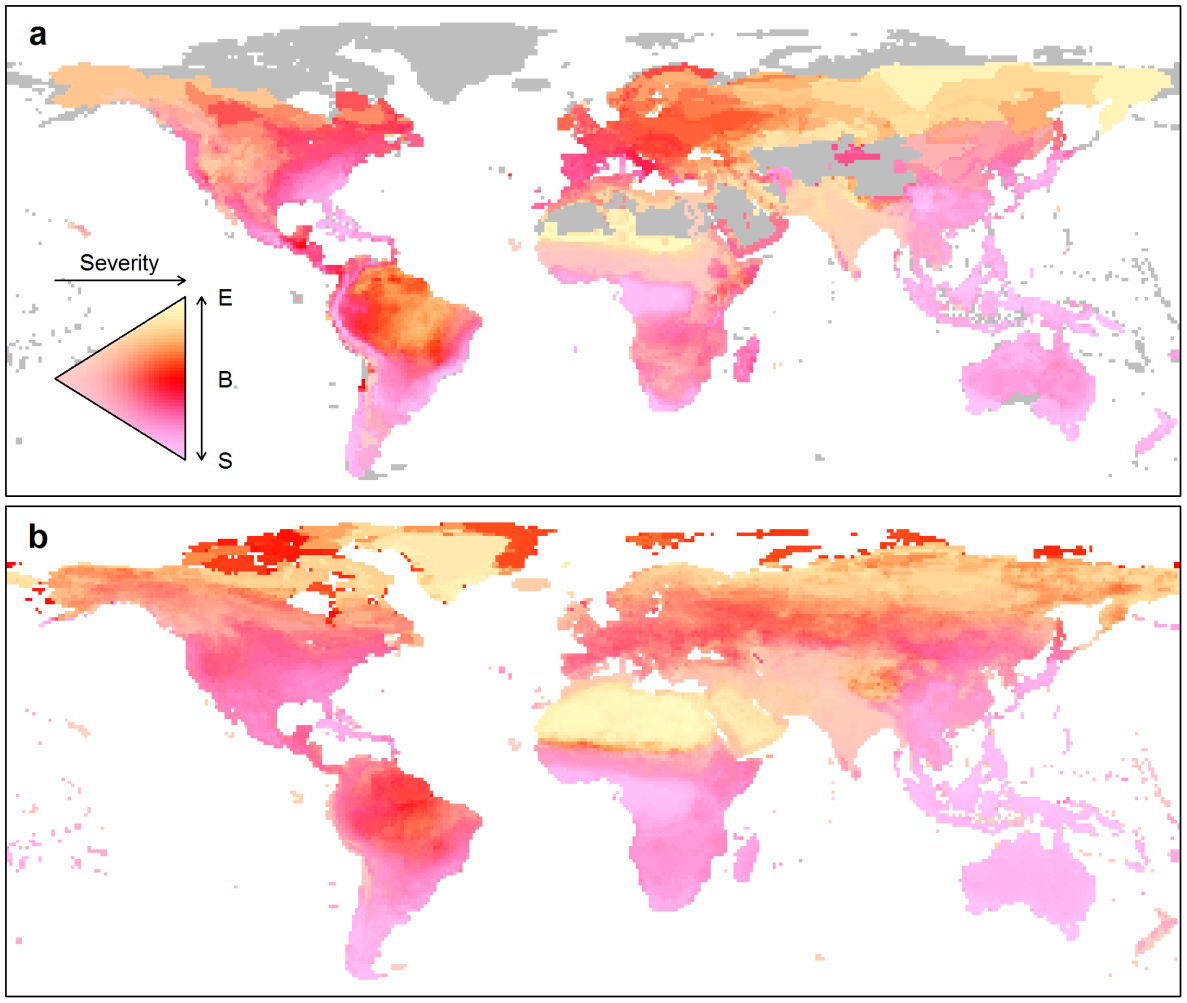
Global patterns of climate breadth and mean climate change. Relative magnitudes of average climate breadth and exposure values within cells for amphibians (a) and mammals (b). As exposures were not qualitatively different across RCPs, only the results for RCP 4.5 are shown. Species in yellow areas are predominantly exposed (E) to climate change and species in magenta areas are predominantly sensitive (S) to climate change, having narrow climate breadth. Red areas are characterised by communities in which both (B) high exposure and high sensitivity may be found. Geographical patterns in sensitivity and exposure are not congruent. Extinction risk may be low in regions where exposure is high where species in those regions are not sensitive to climate change (yellow areas). Similarly, extinction risk may be low in regions that are highly sensitive to climate change where species in those regions are not exposed (magenta areas). Extinction pressure is likely to be highest in those areas where high values of sensitivity and exposure occur together (B - red areas). The saturation of the colours indicates the overall severity of conditions in each cell. Mean cell values were calculated and maps were generated in R^34^.

To remove covariance between the six climate variables, principal components analysis (PCA) was performed to identify six orthogonal axes of variation (Figure 1). Data were transformed where necessary to normalise their distributions (natural log: geographic range size; ^3^√: precipitation of warmest quarter and annual precipitation) and then centred and scaled to standard normal to remove scaling effects before PCA was performed. Sensitivity and exposure analyses then used the value of the six principal components (PCs) within cells.

### Measures of sensitivity and exposure

We base our assessment of sensitivity on the volume occupied by a species' range within the climate space, where a large volume indicates low sensitivity. We found the range of values for each PC within each species' geographic range (Figure 1a, 1b) and took the geometric mean of those ranges to provide a normalised measure of volume. Our method is broadly similar to BOXCAR^31^, but does not place any reliance on the numerical values of climate variables to define an envelope of suitable climate. Rather we use the volume in climate space as a measure of the breadth of climate a species experiences. We refer to this measure as “climate breadth”.

We measured exposure as the mean Euclidian distance between current and future (2050) grid cell values in climate space (Figure 1c). The axes of the current global climate space were interpolated over values for predicted future climate using the transformation, centre and scaling parameters for current conditions. We used climate predictions from 11 GCMs and four relative concentration pathways (RCPs) and used the mean species' exposure value across the different GCMs in our analyses for each RCP^32^.

The dataset from IUCN described ranges for 6156 species of amphibians and 5227 species of mammals. Species were excluded from the analysis where: they were listed as extinct or extinct in the wild; there was taxonomic mismatch between IUCN Red List and distribution data; their entire range occurred in a single 1° grid cell, as variation in climate variables would be zero; and where current and/or future climate data were unavailable for their geographic ranges. Following these exclusions, sensitivity and exposure were calculated for 4713 amphibian and 4923 mammalian species.

We defined broad categories of relative sensitivity to climate change based on percentiles of the distribution of climate breadths. Species with climate breadths less than the percentile value were deemed “sensitive” and species with exposure values greater than the percentile value were deemed to be exposed. We varied the percentile applied in the definition of sensitive and exposed species from 10% to 50% to explore the robustness of results to the threshold.

For each taxonomic group, we generated global maps of grid cell average values of climate breadth and exposure across the species in each grid cell, as well as maps of sensitive and exposed species richness. Richness was corrected for cell size (sensitive or exposed species per km^2^) to account for latitudinal variation in cell sizes. We explored the level of congruence between sensitive and exposed species and between taxonomic groups. Congruence was assessed by Pearson correlation coefficients among grid cells. To account for spatial autocorrelation, significance values were based on the estimated effective sample size (*ess*) for the degree of spatial non-independence observed^33^.

All analyses were conducted in the R statistical environment^34^.

## Author Contributions

M.G.D. and G.M.M. designed the research. M.G.D. carried out the research. C.D.L.O. provided R code for some analyses. M.G.D., C.D.L.O., K.B.S. and G.M.M. wrote the paper.
